# Innate antimicrobial immunity in the skin: A protective barrier against bacteria, viruses, and fungi

**DOI:** 10.1371/journal.ppat.1007353

**Published:** 2018-12-06

**Authors:** Margaret Coates, Sarah Blanchard, Amanda S. MacLeod

**Affiliations:** 1 Department of Dermatology, Duke University, Durham, North Carolina, United States of America; 2 Department of Immunology, Duke University, Durham, North Carolina, United States of America; 3 Pinnell Center for Investigative Dermatology, Duke University, Durham, North Carolina, United States of America; Geisel School of Medicine at Dartmouth, UNITED STATES

## Innate immunity is an essential defense against pathogens

The epidermis, the outermost layer of the skin, is a physical barrier against pathogens. However, breach of the skin barrier through wounding introduces a myriad of microbes to the site of injury. Upon disturbance of the epidermal barrier, the innate immune system and its effectors play a key role in protecting humans against cutaneous and systemic infection [1]. Major constituents of the innate immune system include phagocytic cells, such as macrophages, neutrophils, and dendritic cells, as well as innate leukocytes, such as natural killer (NK) cells, mast cells, basophils, and eosinophils. In addition, epidermal keratinocytes act as active innate immune cells. In response to sensing pathogen-associated molecular patterns (PAMPs) expressed by microbes and host danger molecules, innate immune receptors present on keratinocytes become activated, causing release of inflammatory cytokines and host antimicrobial molecules [2, 3].

## Recognition of pathogens

**Fig 1:**
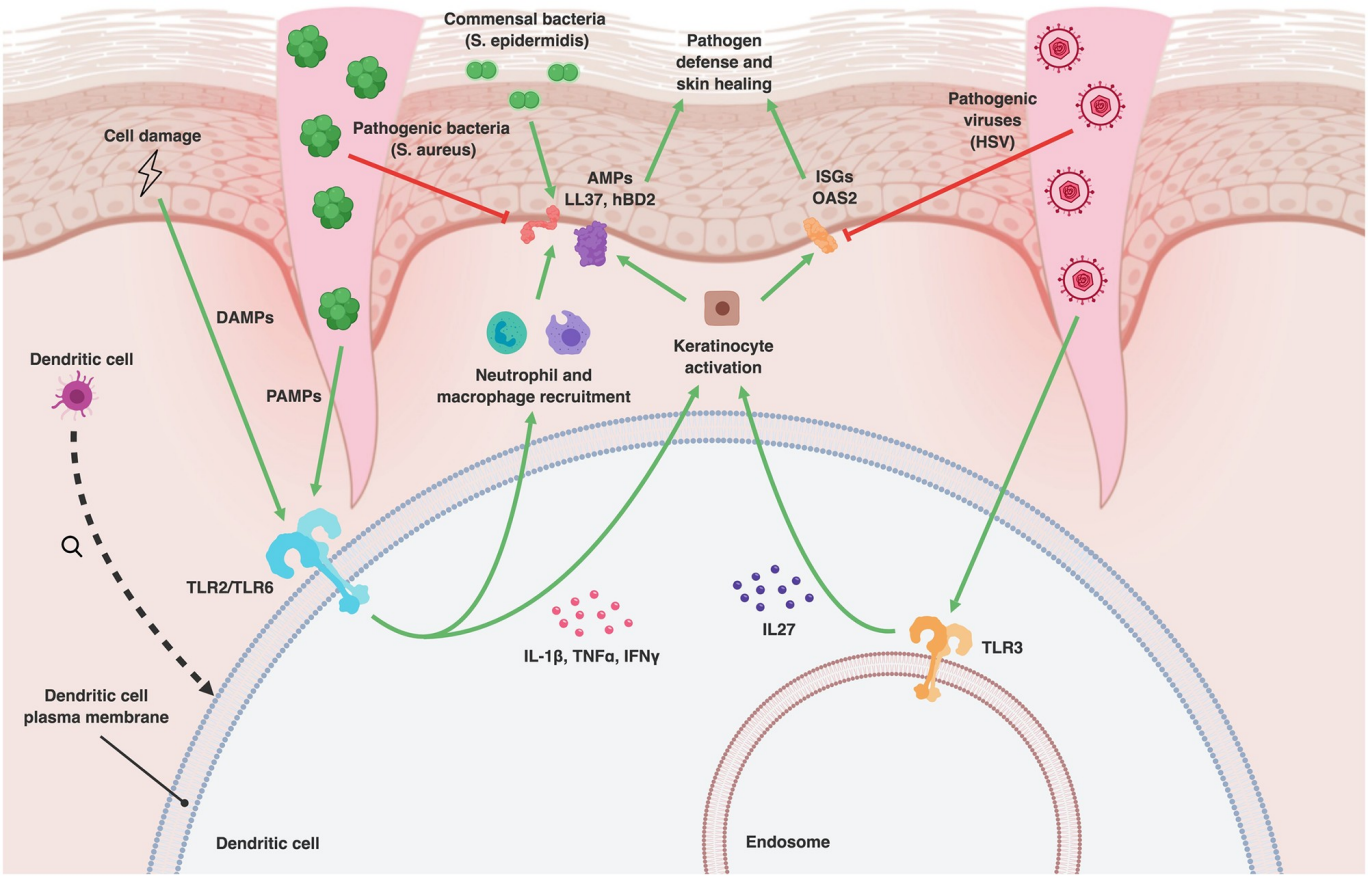
Antimicrobial and antiviral signaling in the skin. * Skin injury allows pathogenic bacteria and viruses to penetrate the epidermis. PAMPs and DAMPs are recognized by TLRs, which induce the projection of pro-inflammatory cytokines by dendritic cells. Examples of pro-inflammatory cytokines include IL-1β, TNFα, and IFNγ. Inflammatory cytokines recruit neutrophils and macrophages to the site of injury and promote production of AMPs by these inflammatory cells and also by keratinocytes. The AMPs produced include cathelicidins (LL-37) and defensins (hBD2). Some TLRs, such as TLR3, recognize viral components. IL-27 is produced in response to TLR3 activation and induces translation of anti-viral ISGs, such as OAS2, by keratinocytes. AMPs and ISGs are important effector molecules for pathogen defense and skin healing. Commensal bacteria, such as *Staphylococcus epidermidis*, promote AMP production. Pathogenic bacteria, such as *S*. *aureus*, inhibit the production of AMPs; pathogenic viruses, such as HSV, inhibit ISG production. *Created with BioRender. AMP, antimicrobial protein; DAMP, damage-associated molecular pattern; hBD2, human β-defensin-2; HSV, herpes simplex virus; IL, interleukin; IFN, interferon; ISG, interferon-stimulated gene; OAS, oligoadenylate synthetase; PAMP, pathogen-associated molecular pattern; TLR, toll-like receptor; TNF, tumor necrosis factor.

The first step of any immune response is recognition of potential pathogens. Germline-encoded pattern recognition receptors (PRRs) recognize PAMPs present on microbes and damaged-associated molecular patterns (DAMPs) on host cells (Fig 1) [4]. The four primary groups of human PRRs are toll-like receptors (TLRs), nucleotide-binding oligomerization domain-like receptors (NLRs), retinoic acid-inducible gene 1 (RIG-I)-like helicase receptors (RLRs) and c-type lectin receptors (CLRs) [4]. Signaling through PRRs has long been known to be essential for activation of the innate immune response. For example, stimulation of TLR2 increases the immune response to pathogens and helps rescue the inflammatory response of immunosuppressed patients with sepsis [5]. Although PRRs are not as specific as immune effectors of the adaptive immune system, different PRRs have evolved to recognize different molecular patterns [6]. For example, TLR2, TLR6, and nucleotide-binding oligomerization domain-containing protein 2 (NOD2) appear to play an important role in host defense against staphylococcal aureus, whereas TLRs 2, 3, 7, 8, and 9 have been found to be activated by many viruses, including members of the herpesviruses, papillomaviruses, and poxviruses [7, 8]. CLRs and TLRs 2, 4, and 9 are thought to be primary receptors involved in recognition of fungal pathogens such as *Candida albicans*, and there are reports of specific PRR deficiencies in patients with chronic mucocutaneous infections [9, 10].

## The inflammatory cascade

Activation of PRRs leads to initiation of the inflammatory cascade. Soon after hemostasis, adhesion molecules are expressed in response to pro-inflammatory cytokines, such as interleukin-1β (IL-1β), tumor necrosis factor-α (TNF-α), and interferon-γ (IFNγ) [11]. Polymorphic neutrophils are recruited by CXC chemokines containing asparagine-leucine-arginine (ELR) motifs, such as IL-8, which transmigrate across capillary walls and interact with adhesion molecules [12]. Neutrophils begin phagocytosis of pathogens and tissue debridement. Later during inflammatory cascade, macrophages are the predominant immune cell type. Macrophages of the pro-inflammatory phenotype (M1) continue phagocytosis as well as amplify the inflammatory response. Anti-inflammatory cytokines, such as transforming growth factor β (TGFβ), cause inflammatory M1 macrophages to shift to an anti-inflammatory phenotype (M2), which promotes wound repair and closure [12].

## Endogenous antibacterial and antiviral proteins help the innate immune system fight pathogenic organisms

Antimicrobial proteins (AMPs) are produced by keratinocytes, infiltrating immune cells, and skin commensal organisms and provide antibiotic-like protection for the skin. AMPs are directly bactericidal through cell lysis, with a preference for prokaryotic cell membranes [13]. Other antimicrobial mechanisms include inhibition of bacteria protein and DNA synthesis. Antifungal mechanism of AMPs involve disruption of the fungal mitochondrial membrane [13]. Two families of AMPs, cathelicidins and defensins, are illustrative of the potent activity of these proteins. Cathelicidins and defensins provide broad-spectrum protection against gram-positive and gram-negative bacteria [14]. For example, the human cathelicidin LL-37 has potent direct antibacterial activity against bacteria such as Group A *Streptococcus* (GAS), and mice deficient in cathelicidins have been shown to have higher susceptibility to GAS infection [15]. Decreased expression of human cathelicidin LL-37 and human β-defensin-2 (hBD-2) in lesions of atopic dermatitis compared to amplified expression in psoriatic lesions may help account for the increased susceptibility to secondary bacterial infection that is seen in patients with atopic dermatitis but not psoriasis [16]. Some defensins, including hBD1, are expressed constitutively in epithelial cells [14], whereas others, such as hBD2, are only constitutively expressed at very low levels, with a dramatic increase in their production during inflammation [17]. One AMP, dermcidin, is released by sweat glands and displays broad antimicrobial activity, demonstrating a role for sweat in microbial protection [18]. In addition to direct activity against pathogens, AMPs also have immunomodulatory activity. For example, LL-37 induces chemotaxis of neutrophils, monocytes, mast cells, and T cells. LL-37 levels are also noted to be dramatically lower in chronic ulcers than in wounds that undergo normal healing, which highlights the importance of this AMP in wound healing, in addition to innate immunity [19]. hBD2 displays chemokine-like activity to attract dendritic cells and memory T cells and also promotes histamine release by mast cells [14]. Finally, regenerating islet-derived protein 3A (REG3A), an AMP present in the gut as well as the skin, promotes keratinocyte proliferation, suggesting a role for AMPs in wound healing as well as antimicrobial defense [20].

PRRs also recognize viral components, leading to transcription of antiviral interferon-stimulated genes (ISGs). One example of an ISG is oligoadenylate synthetase (OAS). OAS identifies viruses through binding of viral double stranded RNA (dsRNA) and activation of an intracellular latent RNase (RNase L), which leads to the degradation of viral RNA [21]. OAS proteins also function through an RNase L−independent mechanism in which proteins that are released from virus-infected cells act extracellularly as an antiviral agent through paracrine signaling [22]. Recent discoveries have highlighted the importance of IL-27 signaling in wound healing and ISG responses [23]. ISGs become strongly up-regulated in epidermal keratinocytes following stimulation with recombinant IL-27, and mice lacking the IL-27 receptor have been found to have delayed wound healing.

## How pathogenic microbes counteract immune defenses

*Staphylococcus aureus* is the most common cause of bacterial skin infections. The mechanisms by which it evades eradication by the innate immune system are representative of the strategies employed by other microbes to counteract immune defenses. For example, *S*. *aureus* releases staphylococcal superantigen-like proteins and toxins that prevent neutrophil migration and cause neutrophil lysis [24]. A second evasion strategy involves the release of membrane vesicles that contain factors that inactivate the complement system. [25]. One such factor is Staphopain A, which cleaves elastin and inactivates C-X-C motif chemokine receptor 2 (CXCR2) and C5b-complement [26]. *S*. *aureus* also produces a metalloproteinase called aureolysin that cleaves and inactivates the AMP LL-37 [27]. Another protease, staphylokinase, undergoes complex formation with human defenses, leading to their inactivation [28]. *S*. *aureus* is also able to alter its hydrophobicity through production of a surface protein, iron-regulated surface determinant protein A (IsdA), resulting in resistance to hBD2 and LL-37 [29].

Viruses that affect the skin also have virulence factors that aid in evasion of the innate immune system. Production of IFN-α and IFN-β is decreased in skin samples infected with herpes simplex virus 2 (HSV2) [30]. The action of OAS2 is prevented by secretion of Us11 protein by HSV1 [31]. Poxviruses, particularly vaccinia virus, have become particularly adept at evading host immunity through the production of peptides that block activation of TLRs [32] Other viruses and pathogenic microbes employ similar mechanisms for immune evasion.

## Commensal organisms promote eradication of pathogenic bacteria and viruses and encourage healing

Previous studies have demonstrated that a synergistic relationship between the human host and the commensal skin microbiome promotes successful wound healing and overall health. Skin commensal microbes, such as *S*. *epidermidis*, produce AMPs that act alongside endogenous AMPs produced by human keratinocytes to provide antibiotic-like protection for the skin; *S*. *epidermidis* also enhances the production of AMPs by keratinocytes [2]. One study identified a small molecule produced by *S*. *epidermidis* that activates TLR2 signaling and induces AMP production by keratinocytes [33]. Other peptides produced by *S*. *epidermidis*, such as a group of phenol-soluble modulins, display direct antimicrobial action against pathogenic bacteria, including *S*. *aureus* [34]. *S*. *epidermidis* can even prevent uncontrolled inflammation, a hallmark of chronic wounds [33]. Commensal bacteria also modulate antiviral immunity. Lipoteichoic acid, a cell-wall component of gram-positive bacteria, increases mast cell activity against vaccina viruses [35].

## Too much of a good thing: Immune hyperactivation and microbial superinfection

Excess inflammation underlies multiple common skin pathologies and is detrimental to skin healing. One of the most common skin conditions, acne vulgaris, is characterized by excess production of pro-inflammatory cytokines and dermal inflammation. Inflammation of the skin may even precede infection with *Propionibacterium acnes* [36]. Rosacea is also marked by excess inflammation; TLR2 sensitivity is increased in lesional keratinocytes [37]. Similarly, REG3A, a peptide that promotes wound reepithelialization, inhibits keratinocyte terminal differentiation and promotes keratinocyte hyperproliferation in psoriatic skin [20]. In addition to contributing to the pathogenesis of skin disease, excess inflammation impedes healing by preventing progression into the proliferative phase of wound closure. Excess inflammation is particularly prevalent in diabetic ulcers and venous ulcers.

## Conclusion

The innate immune system is integral to the prevention of skin infection and eradication of pathogenic bacteria and plays an essential role in skin healing. Recognition of bacteria and viruses initiates the inflammatory cascade involving the release of cytokines, recruitment of immune cells, and production of AMPs and ISGs. AMPs and ISGs represent one of the most important and robust immune mechanisms in the skin. However, pathogenic bacteria—such as *S*. *aureus*—and cutaneous viruses have evolved mechanisms to counteract innate immune mechanisms. Commensal skin bacteria assist the innate immune system with eradication of pathogens through production of AMPs and by enhancing the activity of innate immune cells. Finally, despite the importance of innate immunity, excess immune activation underlies some cutaneous diseases and is detrimental to wound healing.
